# Droplet Digital PCR for Estimating Absolute Abundances of Widespread Pelagibacter Viruses

**DOI:** 10.3389/fmicb.2019.01226

**Published:** 2019-06-12

**Authors:** Francisco Martinez-Hernandez, Inmaculada Garcia-Heredia, Monica Lluesma Gomez, Lucia Maestre-Carballa, Joaquín Martínez Martínez, Manuel Martinez-Garcia

**Affiliations:** ^1^Department of Physiology, Genetics, and Microbiology, University of Alicante, Alicante, Spain; ^2^Marine Virology Laboratory, Bigelow Laboratory for Ocean Sciences, East Boothbay, ME, United States

**Keywords:** marine, ddPCR, *Pelagibacter ubique*, abundance, virus, single-virus genomics, vSAG 37-F6, HTVC010P

## Abstract

Absolute abundances of prokaryotes are typically determined by FISH. Due to the lack of a universal conserved gene among all viruses, metagenomic fragment recruitment is commonly used to estimate the relative viral abundance. However, the paucity of absolute virus abundance data hinders our ability to fully understand how viruses drive global microbial populations. The cosmopolitan marine *Pelagibacter ubique* is host for the highly widespread HTVC010P pelagiphage isolate and the extremely abundant uncultured virus vSAG 37-F6 recently discovered by single-virus genomics. Here we applied droplet digital PCR (ddPCR) to calculate the absolute abundance of these pelagiphage genotypes in the Mediterranean Sea and the Gulf of Maine. Abundances were between 360 and 8,510 virus mL-1 and 1,270–14,400 virus mL-1 for vSAG 37-F6 and HTVC010P, respectively. Illumina PCR-amplicon sequencing corroborated the absence of ddPCR non-specific amplifications for vSAG 37-F6, but showed an overestimation of 6% for HTVC010P from off-targets, genetically unrelated viruses. Absolute abundances of both pelagiphages, two of the most abundance marine viruses, suggest a large viral pelagiphage diversity in marine environments, and show the efficiency and power of ddPCR to disentangle the structure of marine viral communities. Results also highlight the need for a standardized workflow to obtain accurate quantification that allows cross data comparison.

## Introduction

Ecosystems harbor an immense abundance of viruses. Although they probably infect all marine forms of life, the vast majority of them are bacteriophages, i.e., viruses that infect bacteria. An estimated standing stock of approximately 10^30^ viral particles are contained in global oceans (Suttle, 2007). With 10^23^ infections every second, they are responsible for the mortality of up to 50% of microbial populations per day (Weinbauer, 2004), and consequently exert a significant influence on microbial population dynamics and on global biogeochemical cycles through the “viral shunt” (Lara et al., 2017).

The conserved universal marker gene 16S rRNA in bacteria (Pace, 1997; DeLong and Pace, 2001) has enabled the use of *in situ* fluorescence hybridization (FISH) for absolute quantification of important taxa. The absence of conserved molecular marker genes in viruses (Edwards and Rohwer, 2005) complicate this approach. Instead, *in silico* metagenomic fragment recruitments have been widely used to estimate relative abundances of marine viruses (Mizuno et al., 2013; Zhao et al., 2013; Roux et al., 2016, 2017; Martinez-Hernandez et al., 2017). However, quantification of specific ecologically relevant viruses, in particular, those that infect abundant marine bacteria such as Pelagibacteriaceae, is key to understanding viral assemblages structure (Baran et al., 2018), distribution and ecological impact. The widespread Pelagibacteriaceae (alphaproteobacteria) are distributed throughout the oceans and represent 15–60% of bacterioplankton (Morris et al., 2002; Malmstrom et al., 2005; Eiler et al., 2009). A few years ago, four pelagiphages were isolated and discovered to be highly represented in marine viromes. In particular, strain HTVC010P was found to be the most abundant among those (Zhao et al., 2013). More recently, a novel methodology based on single-virus genomics have unveiled the genome of the single-virus vSAG 37-F6, that possibly represents the most abundant viral population in the marine surface virosphere (Martinez-Hernandez et al., 2017). This methodology, in contrast to viral metagenomics (i.e., collection of all viral genetic material), allows to obtain the genome from single uncultured viruses one at a time by combining fluorescence-activated virus sorting (FAVS), whole-genome amplification and sequencing of individual sorted viruses from marine viral samples. In a follow-up study, several 37-F6-like viruses were found within single-sorted *Pelagibacter* cells (Martinez-Hernandez et al., 2018). Thus, vSAG 37-F6 virus represents the putative most abundant and widespread uncultured pelagiphage population with a vast unrecognized genomic microdiversity (Martinez-Hernandez et al., 2017, 2018), and a high *in situ* viral activity in coastal marine samples (Alonso-Sáez et al., 2018).

Absolute and precise quantification of specific viruses will allow us to improve our understanding and parameterization of their contribution to the “viral shunt” and to biogeochemical cycles, as well as decipher specific virus-host interactions through abundance correlation. In recent years, new culture-independent tools have been implemented to quantify specific viral groups from environmental samples based mainly on phage-FISH (Allers et al., 2013) and polymerase chain reaction (PCR) techniques, such as quantitative PCR (qPCR) (Eggleston and Hewson, 2016) or PCR polony method (Baran et al., 2018). For instance, qPCR has been used to quantify two pelagiphage isolate strains in the South Atlantic using specific primers (Eggleston and Hewson, 2016). Likewise, the PCR polony method has been reported to effectively quantify heterogeneous virus (cyanophages) populations using degenerate primers and probes (Baran et al., 2018). Droplet digital PCR (ddPCR) is becoming increasingly used in clinical virology for the investigation of human pathogenic viruses within patients (Strain et al., 2013; Kiselinova et al., 2014; Schwartz and Lowen, 2016; Trypsteen et al., 2016; Vynck et al., 2016) and in environmental samples (Rački et al., 2014; Sedji et al., 2018). For instance, recently, in sewage-impacted urban waterway, this technology has been employed to monitor the absolute abundance of the putative most abundant phage in wastewater, the virus CrAssphage, showing values from 10 to 1,600 viruses per mL (Stachler et al., 2019).

In ddPCR, the mastermix-template mixture is initially partitioned into up to several million water-oil emulsion (droplets) prior to thermocycling, such that independent reactions occur within each droplet (Vynck et al., 2016). Significant advantages of this approach over other PCR dependent methods include: independence of a calibration curve to determine the copy number of the target sequence, physical separation of inhibitors from target DNA molecules within individual droplets (Vynck et al., 2016), higher precision and accuracy, and absolute quantification, compared to relative quantification of targets based on comparisons to standard curves from template dilutions (Pinheiro et al., 2012; Hall Sedlak and Jerome, 2014; Kiselinova et al., 2014; Bosman et al., 2015). In particular, ddPCR is more appropriate for quantifying specific viral species than PCR polony, which performs better for viral groups that include different species or genera (Baran et al., 2018).

In this study, we employed ddPCR to calculate the number of viral particles of two abundant and cosmopolitan pelagiphages, the uncultured vSAG 37-F6 and the cultured isolate HTVC010P, in Western Mediterranean and Gulf of Maine coastal surface seawater samples. Both pelagiphages infect abundant *Pelagibacter* spp. and consequently, are likely to contribute significantly to the global marine biogeochemical cycles.

## Materials and Methods

### Marine Sample Collection and Processing

Mediterranean surface seawater samples (25 L each) for the ddPCR analysis were collected from the Western Mediterranean Sea at Blanes Bay Microbial Observatory (BBMO) (41° 40′ 13.5″ N, 2° 48′ 0.6″ E; 2.7 miles offshore) on May 10, 2017 and at Cape Huertas (Alicante coast, 38° 21′ 14.3″ N, 0° 25′ 36.6″ W) on July 6, 2017. A Gulf of Maine coastal surface water sample (5 L) was collected at the Bigelow Laboratory for Ocean Sciences’ dock (69° 34′ 35.6″ N, 69° 34′ 41.5″ W) on July 7, 2017. Seawater was sieved through a 20 μm mesh, and filtered through a 0.22 μm membrane filter. Then, viruses were concentrated to 20 mL using tangential flow filtration (TFF) with Vivaflow 200 membrane (Sartorius) for Mediterranean Sea samples (BBMO and Cape Huertas) and QuixStand benchtop system cross-flow hollow fiber system equipped with a 300 kDa NMWC Xampler^TM^ cartridge (GE Healthcare) for the Gulf of Maine sample. The concentrated samples were filtered again through a 0.22 μm membrane to ensure removal of cellular organisms, which was corroborated by epifluorescence microscopy by SYBR Gold staining of these viral concentrated fractions. Thus, in viral concentrates of this study, *Pelagibacter* cells and infecting viruses were efficiently excluded from our ddPCR results. Next, these viral fractions, free of cells, were further concentrated to 1 mL using Amicon® Ultra-15 centrifugal filters (100 kDa-cut off, ref. UFC910008, Millipore). During the filtration and TFF process, VLPs are lost and retained in the membrane. To estimate the loss factor of VLPs at each step, a subsample was collected for flow cytometry enumeration to determine potential virus particle loss factor throughout sample processing. This loss factor (defined as the quotient between the values of viral concentration in the natural collected seawater sample and in the ultraconcentrated viral sample) can be later used to mathematically correct the ddPCR data results, if needed, to accurately determine the VLP mL^–1^ within the original unfiltered seawater sample.

Nucleic acids were extracted from the final 1 mL concentrate using commercial kits QIAamp® Ultrasense Virus (Cat. No. 53704, QIAGEN) or MasterPure^TM^ Complete DNA & RNA Purification (Cat. No. MC85200, Epicenter), according to the manufacturer’s protocols, applying a previous extracellular DNase treatment using 5 U of Turbo DNase I (Ambion) according to manufacture’s protocol for 1 h at 37°C.

Seawater samples for Illumina amplicon sequencing included the Cape Huertas sample above mentioned, and two additional samples (100 L each) collected from the Mediterranean Sea during the *REMEI* expedition, at the surface (5 m depth, 40° 29′ 15.6″ N, 3° 3′ 45.8″ E) and at the deep chlorophyll maximum (DCM, 84 m depth, 40° 29′ 29.4″ N, 3° 3′ 15.3″ E) on September 27, 2017 and September 29, 2017, respectively. The *REMEI* expedition samples were processed and DNA was extracted as described for the Cape Huertas samples above.

### Viral Abundance

Virus-like particles (VLP) abundance from the marine samples used for ddPCR were enumerated by flow cytometry following the reference protocol (Brussaard, 2004), using SYBR Gold dye instead of SYBR Green I. Flow cytometry analyses were performed with a FACS Canto II cytometer (BD Biosciences) equipped with a 488-nm laser. Green fluorescent detector voltage was set to 525 and a threshold on green fluorescence was set at a value of 200. Green fluorescence, total counts and side scatter were recorded for each sample and blank.

### Viral Fragment Recruitment

*In silico* abundance of vSAG37-F6 and HTVC010P was estimated by virome fragment recruitment of these viruses using BBMO (Martinez-Hernandez et al., 2017), *Tara* expedition (Brum et al., 2015), Pacific Ocean Virome (Hurwitz and Sullivan, 2013) and Sargasso Sea (Angly et al., 2006) viromes. Fragment recruitment was performed as described in Martinez-Hernandez et al. (2017), considering only hits with ≥ 95% of nucleotide identity.

### Primers and Probe Design for ddPCR

A probe and primer set for the vSAG 37-F6 (37-F6 ddSeq4; Fw: TGTGTACCTTCACCCACTTG, Rv: AGAACCATCAGGAA CTCTGTTAC, Pb: TGACCAGCTTGAACCACAATACCCA) was designed and its specificity was checked as described in Martinez-Hernandez et al. (2018) (Supplementary Figure 1) adding the 4 putative vSAG 37-F6-like pelagiphages obtained from single-amplified genomes (SAGs) to the custom viral database (Martinez-Hernandez et al., 2018). These primers and probe targeted a gene encoding the specific hypothetical protein HPX (818 amino acids) of the vSAG 37-F6 genome (for more details see Figure 4 of a previous study in Martinez-Hernandez et al., 2017). Available reported primers and probe for pelagiphage HTVC010P (10P ddSeq1; Fw: GAAATGCAACAGATGCAACA, Rv: TG CTTCTTCTGGCAATGCT; Probe: GCAGGAGGAGATATAG CACCACTAGCG) (Eggleston and Hewson, 2016) were used here to compare the abundance of both viruses.

### Pre-droplet Digital PCR Optimization

Prior to ddPCR, Taqman probes (labeled with TET fluorophores (IDT); green fluorescence) and primers were tested by standard PCR and qPCR, using multiple-displacement amplification (MDA) product of the virus vSAG 37-F6 and DNA extracted from BBMO sample as templates. The BBMO DNA originated from the same seawater sample from which vSAG 37-F6 was recovered (Martinez-Hernandez et al., 2017). The PCR reactions were generated using 1.5 ng of template, 450 nM each of forward and reverse primers and 1× of REDTaq® ReadyMix^TM^ PCR Reaction Mix (Sigma-Aldrich). Thermal cycling conditions were: initial denaturation at 94°C for 4 min, followed by 35 cycles of 20 s at 94°C, 30 s at 55°C and 1 min of 72°C, and a final extension of 30 min at 72°C. Amplicons were visualized on a 1.5% agarose gel (TAE 1×) to verify the correct length of the amplicon and the absence of non-specific products. Finally, PCR products were purified with a MinElute® Reaction Cleanup kit (QIAGEN) and were Sanger sequenced in a Genetic Analyzer ABI PRISM 3130XL (Applied Biosystems) to check that the target region was properly amplified, and that no other non-specific products had been obtained. Then, qPCR was used to test the designed probes. The qPCR conditions were as follows: 1× TaqMan® Genotyping Master Mix (Life Technologies), 450 nM of each forward and reverse primers, 200 nM of target probe, and 2 μL of the diluted positive control template. Thermal cycling conditions were an initial denaturation step at 95°C for 10 min, followed by 55 cycles at 95°C for 15 s, and 60°C for 1 min. The reactions were carried out using a CFX96 Real-Time PCR Detection System (Bio-Rad). The reactions were optimized as previously described (Gilg et al., 2016).

### Droplet Digital PCR Quantification

Droplet digital PCR reactions were conducted using a RaindDrop® digital PCR system (RainDance Technologies). Reaction mix partitioning of droplets was conducted in the RainDrop Source (RainDance Technologies). ddPCR involves three steps: (1) individualization or partition of the nucleic acid viral sample and PCR reagents in up to 5 million picoliter-sized (5 pL) droplets (each droplet encapsulates a single target molecule), (2) PCR amplification of droplets, and (3) detection of positive droplets based on fluorescence signal. Twenty five microliter final volume ddPCR reactions were set up, as for qPCR. ddPCR conditions were as follows: 1 × TaqMan® Genotyping Master Mix (Life Technologies), 450 nM of each forward and reverse primers, 100 nM TaqMan probe for vSAG 37-F6, while 250 nM TaqMan probe concentration was used for HTVC010P. Optimal probe concentrations were determined as described in Gilg et al. (2016). Briefly, both probes were tested, separately and together, at different concentrations, until two discriminated and defined regions were clearly detected at different fluorescence levels of the *Y* axis (TET fluorescence; Supplementary Figure 2). Probe concentrations were defined and optimized with different dilutions of pure DNA template from MDA product of vSAG 37-F6 (Martinez-Hernandez et al., 2017). In this ddPCR equipment, as per manual recommendations, the different TaqMan probe concentration targeting different sequences (in this case vSAG 37-F6 and HTVC010P) allows to define and distinguish each viral population in the ddPCR plot. As we used the higher TaqMan probe concentration (TET labeled) for the HTVC010P, then higher fluorescence intensity is obtained for this population, and thus positive droplets of HTVC010P appeared upper in the *Y* axis of the ddPCR plot (Green TET fluorescence). Conversely, for 37-F6, as lower concentration of TaqMan probe (TET labeled) was employed, the positive droplets of this population are then displayed lower in the *Y* axis. The amount of environmental viral DNA added to 25 μL of ddPCR mix reaction differed for each sample to allow that negative droplets were, as per manufacture’s protocol of ddPCR equipment, at least, 98.5% of the total intact droplets, which ensures that only one DNA target molecule is encapsulated in each droplet. With these samples, we used 0.143, 0.092, and 4.550 ng of extracted viral DNA from BBMO, Cape Huertas and the Gulf of Maine, respectively.

**FIGURE 1:**
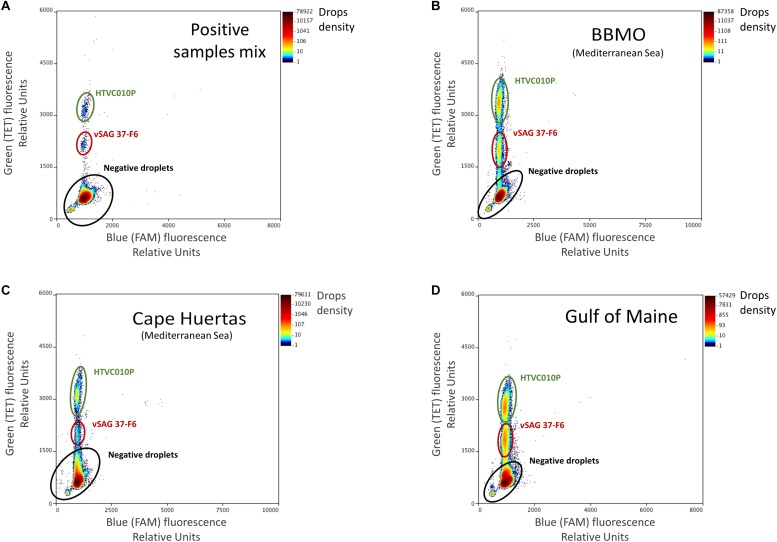
Droplet digital PCR (ddPCR) plots. Fluorescence levels of TET-labeled probes were obtained and regions with a high density of points in the TET axis (*Y*-axis), gated with colored ovals, represent the populations of each target virus. Note that the gate corresponding to vSAG 37-F6 was delimited at a lower level in the TET axis than that of the HTVC010P population due to the difference in concentration of each probe (for vSAG 37-F6, 100 nM, while it was 250 nM for HTVC010P). The panels represent different sample points, **(A)** control: an artificial mix of purified positive PCR amplicon reactions from vSAG 37-F6 and HTVC010P. These two positive PCR reactions were also run separately to corroborate the location of each virus in the plot, **(B)** Blanes Bay Microbial Observatory (BBMO) (Mediterranean Sea), **(C)** Cape Huertas (Mediterranean Sea), and **(D)** Gulf of Maine samples. Negative droplets (no DNA template encapsulated) showing basal fluorescence are indicated as a black elipse.

Amplification was carried out in a C1000 Touch deep well thermal cycler (Bio-Rad), with the following thermal protocol: an initial denaturation at 95°C for 4 min, followed by 40 cycles of 30 s at 94°C, 1 min at 62°C with a ramping rate of 0.5°C sec^–1^, 98°C for 10 min and hold at 4°C. Absolute quantification of target viruses was obtained by the enumeration of fluorescent droplets (positive reactions) on the RainDrop Sense (RainDance Technologies). The data output (positive and negative droplet reactions) was analyzed in RainDrop Analyst II (V1.1.0) and displayed in 2-dimensional scatter plots (Figure 1). Software allowed the correct identification of each viral population, and it was used to apply the spectral correction for each set of reactions, prior to enumerating the intact droplets.

To estimate the absolute abundances of the targeted dsDNA viruses in the original sample, ddPCR quantification values have to be corrected for each sample as follows according to the amount of DNA used in the ddPCR reaction mix and the total theoretical amount of DNA from each viral sample. The latter is directly dependent upon the natural viral concentration (VLPs/mL) previously calculated by flow cytometry. For this estimation, we assumed the widely accepted reference values of a mean viral genome length of 50 kb (Wommack et al., 1999; Steward et al., 2000; Roux et al., 2016), and the universal reported average mass factor of 1.08^–12^ ng for a DNA base pair (average weight of a base pair (bp) is 650 Da; and 1 Dalton is equivalent to 1.67 × 10^–24^ g). Finally, the resulting corrected absolute abundance value from each sample has to be normalized according to the total volume of collected seawater. Very frequently, because of different reasons, different seawater volumes are sampled for each sample. Therefore, corrected and standardized viral abundance is calculated for each target virus using the following formula:

$$\mathrm{Absolute abundance}\;(\mathrm{virus}/\mathrm{mL})=\frac{\mathrm{ddPCR abundance}\;(\mathrm{virus})\times \mathrm{total original DNA}\;(\mathrm{ng})}{\mathrm{DNA template in the ddPCR mix}\;(\mathrm{ng})\times \mathrm{total collected seawater}\;(\mathrm{mL})}$$

This method is detailed in Supplementary Material (Supplementary Data Sheet 1) where we provide an example of calculation for one of the samples used.

Every sample for ddPCR was quantified by triplicate, mean value and standard error were provided, and differences between vSAG 37-F6 and HTVC010P abundances from each sample were compared using a paired sample *t*-test, after check normality (*ks*-test) and homoscedasticity (*F*-test) of every group of data. Statistical analysis was performed using the computing R environment.

### *In silico* Identification of Non-specificities

To check hypothetical non-specific annealing of oligonucleotides (primers and probes) used in ddPCR with other known cultured and uncultured marine viral genomes, primers and probes of each pelagiphage quantified in this study (vSAG 37-F6 and HTVC010P) were compared *in-silico* with a custom viral database. A total of 331,727 viral genomes and genome fragments were considered [40 surface vSAGs (Martinez-Hernandez et al., 2017), 4 putative pelagiphages vSAG 37-F6-like obtained by Single Amplified Genomes (SAGs) (Martinez-Hernandez et al., 2018), 20 viral genomes obtained from single cells (the longest contig for each virus) (Labonté et al., 2015), 1,229 viral fosmids from the Mediterranean Sea (Mizuno et al., 2013, 2016), 179 marine virus isolates available at IMG database, and 330,255 viral contigs from Global Oceanic Virome metagenomic survey (GOV) (Roux et al., 2016) and *Tara* expedition assembly (Coutinho et al., 2017)]. The used viral genome database is available at the public cyberinfrastructure CyVerse (see link to direct download in Supplementary Data Sheet 2 in Supplementary Material). The comparison was carried out using Primer-Blast (Ye et al., 2012) and Blastn with an *E*-value threshold of 10^–5^ optimizing the search for short sequences using the following commands: ‘*-task “blastn-short” and -dust no.*’ Nevertheless, *in silico* determination of primer/probe specificity is limited by what sequences are available in public databases at the time of the primer/probe design.

Potential non-specificity was considered when the primers and the probe for each pelagiphage (vSAG 37-F6 or HTVC010P) aligned to a viral genome of the database, allowing a maximum of two mismatches per alignment.

For determining the taxonomic relationship between the target and the potential non-specific virus, the average genome nucleotide identity (gANI) was calculated for each one of them using Gegenees software with the following parameters: fragment size = 100 and step size = 50 (Ågren et al., 2012).

### Illumina PCR Amplicon Sequencing From Environmental Samples

Modified ddPCR primers with Illumina specific adapters (37-F6 ddSeq4 Fw: TCGTCGGCAGCGTCAGATGTGTA TAAGAGACAGTGTGTACCTTCACCCACTTG, 37-F6 ddSeq4 Rv: GTCTCGTGGGCTCGGAGATGTGTATAAGAGAC AGAGAACCATCAGGAACTCTGTTAC, 10P ddSeq1 Fw: TCGTCGGCAGCGTCAGATGTGTATAAGAGACAGGAAAT GCAACAGATGCAACA and 10P ddSeq1 Rv: GTCT CGTGGGCTCGGAGATGTGTATAAGAGACAGTGCTTCTTC TGGCAATGCT) were used for PCR on DNA samples from Cape Huertas and from the *REMEI* expedition using the conditions described above. The PCR amplicons were sequenced using Illumina MiSeq platform (pair-end 2 × 300 bp) at the FISABIO Genomics Center (Valencia, Spain). Pair-end overlapping sequences (forward and reverse joined reads) were trimmed with Trimmomatic (Bolger et al., 2014). The quality score was at least Q30 in the 96.8% of resulting bases from trimmed pair-end joined reads. The read size employed in sequencing (2 × 300 bp) was higher than the theoretical target amplicons (99 and 92 bp for vSAG 37-F6 and HTVC010P, respectively) in order not to discard potential larger non-specific amplicons in joined reads. Illumina reads from vSAG 37-F6 and HTVC010P are available as (Supplementary Data Sheets 3 and 4, respectively).

Illumina sequenced amplicons were compared using Blastn (*E*-value threshold 1 × 10^–5^) against its respective theoretical viral target (vSAG 37-F6 and HTVC010P ddPCR amplicon regions) to determine the specificity of primers and probes. In addition, Blastn was run with the same parameters against our custom viral database described above to determine the nearest virus for each sequenced amplicon. Best hit (higher bit-score) was obtained for each amplicon. gANI between vSAG 37-F6 or HTVC010P and the non-specific best-hit viruses assigned to Illumina amplicons were calculated using Gegenees software as we described above.

## Results

### Viral Abundances in Natural Seawater Samples

A total of three surface samples were analyzed using ddPCR. Two coastal surface water samples were collected from the Western Mediterranean Sea at BBMO and at Cape Huertas. The other seawater sample was from the Gulf of Maine (Bigelow Laboratory for Ocean Sciences’ dock). Total VLPs were one order of magnitude higher (1.29 × 10^7^ VLP mL^–1^) in the sample collected from the Gulf of Maine than in the oligotrophic Mediterranean samples (between 1.44 and 3.52 × 10^6^ VLP mL^–1^), as determined by flow cytometry (Supplementary Figure 3). Viral abundance data was within the normal range found in typical ocean surface sites according to trophic water status (Winter et al., 2014). The estimated total viral DNA amount in each original sample were 4,4 × 10^3^, 1,9 × 10^3^, and 3,5 × 10^3^ ng in BBMO, Cape Huertas and Gulf of Maine, respectively.

**FIGURE 2:**
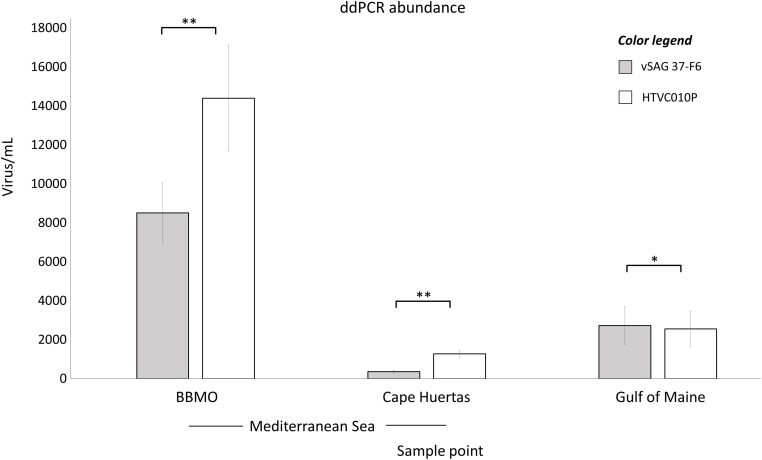
Absolute droplet digital PCR (ddPCR) abundances. The total abundance of vSAG 37-F6 (gray bars) and HTVC010P (white bars) is represented for two Mediterranean sample points, BBMO and Cape Huertas, and for Gulf of Maine sample. The standard deviation value for each quantification (triplicate measurements) is provided. *p*-value of the paired sample *t*-test comparing both viruses within the same sample is shown (^*^p-value < 0.05, ^∗∗^*p*-value < 0.01).

ddPCR results (Figure 2) from BBMO (Mediterranean Sea) showed 14,400 ± 2,740 and 8,510 ± 1,570 virus mL^–1^ for the isolate pelagiphage HTVC010P and vSAG 37-F6, respectively. At Cape Huertas (Alicante, Spain), absolute abundance was 1,270 ± 197 virus mL^–1^ for HTVC010P and 360 ± 72 virus mL^–1^ for vSAG 37-F6. In the Gulf of Maine sample, both viruses showed similar abundances, (HTVC010P: 2,550 ± 903 virus mL^–1^ and vSAG 37-F6: 2,730 ± 973 virus mL^–1^). Statistical significant differences were found between the abundance of both viruses within the same sampling location (BBMO *p*-value: 0.002224, Cape Huertas *p*-value: 0.00627, Gulf of Maine *p*-value: 0.04689). It is worth noting that these abundances represent standing stocks of free virus particles as bacteria cells were detected neither by epifluorescence microscopy nor flow cytometry in viral concentrates (see section “Materials and Methods”).

### *In silico* Abundance of HTVC010P and vSAG 37-F6 Viruses

**FIGURE 3:**
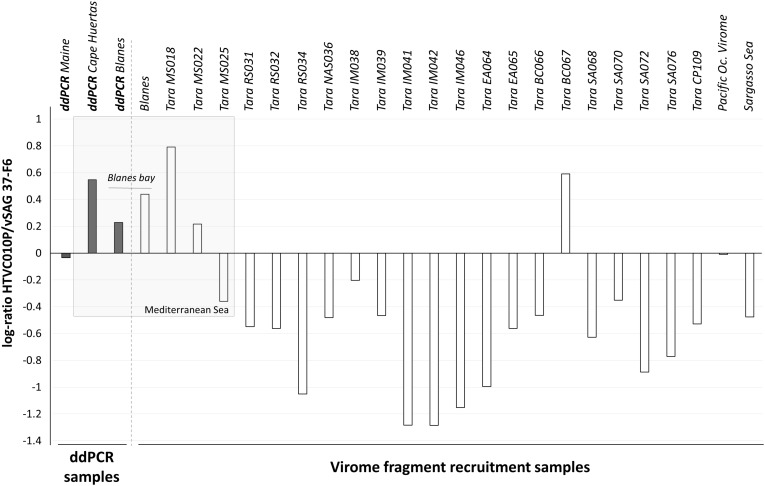
Log_10_-ratio of HTVC010P/vSAG 37-F6 abundances. The log of the resulting quotient obtained from the division of both pelagiphages abundance is represented by each bar. The gray bars represent results obtained by ddPCR experiments and the white bars represent values obtained by virome fragment recruitment data. Positive bars show higher abundance values for the HTVC010P, while negative values indicate a dominance of vSAG 37-F6.

Experimentally determining the absolute abundance of viruses at specific taxonomic levels is necessary for ground-truthing *in silico* virus abundance estimates from metagenomic data. In this study, we performed virome fragment recruitment using marine viromes from all around the world, including one virome originated from the BBMO site included in our ddPCR survey. This analysis showed that for 19 of the 24 viromes obtained from surface water samples around the globe (Brum et al., 2015), vSAG37-F6 was significantly more abundant than pelagiphage HTVC010P (Figure 3). Three of the four viromes where fragment recruitment showed that HTVC010P was more abundant than vSAG 37-F6, were originated from the Western Mediterranean Sea, including the BBMO virome.

### Illumina Sequencing of ddPCR Amplicons

**FIGURE 4:**
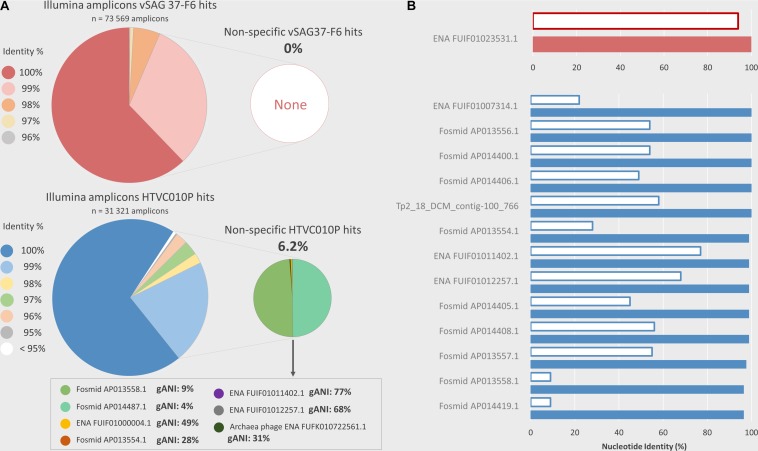
Illumina PCR amplicon sequencing and *in silico* analysis of non-specificities in the ddPCR. ddPCR overestimation, due to potential non-specific viral genomes amplifications, was analyzed using two methods. **(A)** Illumina amplicons sequencing. ddPCR primers were used to sequence amplicons obtained by PCR from natural samples (Cape Huertas and surface and DCM samples from REMEI expedition) using Illumina technology. The nucleotide similarity percentage between Illumina amplicons, which contained the probe region, and the target amplicon (vSAG 37-F6 or HTVC010P) is shown in the large pie shapes diagrams. The small pie chart shows the percentage of amplicons which are more similar to an off-target viral sequence from a custom database (*see section “Experimental Procedures Section”*) than the ddPCR target virus. The complete genome average nucleotide identity (gANI) between HTVC010P and their potential non-specificities are shown in the text box. For vSAG 37-F6 no potential ddPCR non-specificities were found. **(B)** *In-silico* comparison with a custom viral database. Viruses from the custom database, which contain a potentially amplifiable region with ddPCR primers and probes (maximum 2 mismatches for each primer and identical probe) are shown, in red for vSAG 37-F6, and in blue for HTVC010P. The white bars indicate the complete genome ANI % between the target virus (vSAG 37-F6 or HTVC010P) and the potential non-specific viral genome. The colored bars show the nucleotide identity similarity between the target ddPCR amplicon (vSAG 37-F6 or HTVC010P amplicon) and the highly similar region, comprised between primers, in the potential non-specific viral genome.

PCR amplicon sequencing from environmental marine samples with the same primers used for ddPCR corroborated that nearly all droplets counted as positive indeed belonged to the studied viruses. Amplicon read sequences, with identical probe region, were obtained for vSAG 37-F6 (*n* = 73,569) and HTVC010P (*n* = 31,321), respectively. Of these amplicons, 93.6 and 91.4% had ≥ 99% nucleotide identity (100% coverage) with the target sequence regions of HTVC010P and vSAG 37-F6, respectively (Figure 4A). When comparing all amplicon sequences to the entire marine viral database based on best-hit match (the highest bit score), in the case of the vSAG 37-F6 dataset, all sequences had their best match with this virus (always with ≥ 96% nucleotide identity). However, 6.2% of the amplicons obtained with the HTVC010P primers, had the best-hit match to other viruses (Figure 4A), most of them not genetically related with pelagiphage HTVC010P, such as with different marine viral fosmids (Mizuno et al., 2013; Figure 4A). Thus, sequencing data confirmed that the ddPCR signal for vSAG 37-F6 was fully specific, whereas, for the pelagiphage HTVC010P, false positive droplets could be counted, albeit they represented ≈6% of total counts.

## Discussion

### Quantification of the Ubiquitous Marine Viruses vSAG 37-F6 and HTVC010P

In this study, we demonstrate the power and potential of ddPCR to address the absolute abundances of two abundant viral genotypes in the marine viral realm. Specifically, the uncultured cosmopolitan virus 37-F6 discovered by single-virus genomics (SVG) (Martinez-Hernandez et al., 2017), is likely the most abundant marine viral genotype, which together with the viral isolate HTVC010P represent two viruses that have a major contribution on the C marine cycle. Although the abundance and distribution of *Pelagibacter ubique* has been widely studied (Morris et al., 2002; Lefort and Gasol, 2013; Zhou et al., 2018), to our knowledge, only one survey has been carried out to investigate the spatial distribution and abundance of their phages by qPCR (Eggleston and Hewson, 2016), albeit by that time, the uncultured single virus 37-F6 was totally unknown. In that study, through a latitudinal transect in the open Atlantic Ocean, authors obtained a mean abundance of HTVC010P at the surface and DCM of 1.03 ± 2.38 × 10^5^ virus mL^–1^ and 5.79 ± 2.86 × 10^3^ virus mL^–1^, respectively. Data were estimated as a mean of qPCR values obtained from 11 sampling sites along a latitudinal transect of 5,000 km in the open Atlantic Ocean. Most of these samples showed abundance values within the order of 10^3^–10^4^ virus mL^–1^, similar to our ddPCR data. Given the dependence of viruses on their specific hosts, it is likely that the differences observed between this study and our data mirror the distribution of SAR11, which represents a higher percentage (up to 60%) (Morris et al., 2002; Malmstrom et al., 2005; Eiler et al., 2009) of total bacterial community in open oceans (especially at the DCM) than in coastal locations (Lefort and Gasol, 2013). While this is the first study that reports absolute quantification of vSAG 37-F6, based on the similar abundances between HTVC010P and vSAG 37-F6, it is likely that both pelagiphage types have a similar distribution between open and coastal waters.

The other existing PCR-based survey on uncultured marine viral assemblages used the recently developed PCR polony to quantify T7-like cyanophages (Baran et al., 2018). The abundance of this viral population (up to ∼7.70 × 10^5^ VLP) exceeded the estimates for the pelagiphages by ddPCR and qPCR. However, the sequencing of viruses from polonies revealed that each polony was likely comprised of different viral species and genera. Thus, this obtained abundance value is a cumulative abundance data from the entire population comprised of different viruses. In our study, absolute virus abundances are provided at the genotype level.

Previously, relative abundance estimations, obtained by virome fragment recruitment data, showed that both pelagiphages, HTVC010P and vSAG 37-F6, were some of the most abundant and widespread marine viruses (Zhao et al., 2013; Martinez-Hernandez et al., 2017). Nevertheless, by employing either our ddPCR data or the previously reported qPCR results (Eggleston and Hewson, 2016), the cumulative relative abundance of these two pelagiphages compared to the total VLPs (∼1.44 × 10^6^–1.29 × 10^7^ VLP mL^–1^) was less than 1% for all analyzed samples. Interestingly, in the two oligotrophic Mediterranean samples, the overall abundance contribution of both pelagiphages to the total VLP counts was higher (0.11–0.67%) than that for the eutrophic sample from the Gulf of Maine, in which the cumulative abundance of both pelagiphages compared to the total viral community was 0.04%. Thus, these values are much lower than that of *Pelagibacter* spp. compared to the total bacterioplankton (as much as 50%) by means of 16S rRNA gene FISH analysis (Morris et al., 2002), which nevertheless obscure the actual genomic microdiversity and the high number of different *Pelagibacter* genotypes/ecotypes co-existing in the same sample (Vergin et al., 2007; Cameron Thrash et al., 2014; Salter et al., 2015). Recent data (Gregory et al., 2019) have highlighted the high viral microdiversity in temperate and tropical epipelagic waters. Other culture independent studies from a single sample have discovered that the pelagiphage population from a single site is comprised of at least 80 different pelagiphage genotypes (Mizuno et al., 2013), while previous data from single-virus genomics for 37-F6 and HTVC010P combined with metagenomics have unveiled a large “hidden” microdiversity in uncultured pelagiphage population at the strain/species and genus level (Martinez-Hernandez et al., 2017) (Supplementary Figure 4). Furthermore, virome fragment recruitment using more than 6,000 viral contigs and complete uncultured and cultured genomes, showed that in general, the great majority of viruses recruited each as much as 0,1% of viral reads of the viral metagenome, and in some exceptional cases, such as in viral blooms scenarios, this value can increase up to 1% (e.g., Synechococcus phage S-SM2 in NAS036 *Tara* virome sample) or higher (Martinez-Hernandez et al., 2017). It is worth noting that in this study, we have only measured the abundance of only two pelagiphages, that likely represent a tiny fraction of the entire micro-diversity of pelagiphages co-existing in the sample. Thus, data suggest that highly different viral populations of pelagiphages (many yet unknown) co-occur in the analyzed samples, as is the case for other abundant phages and eukaryotic viral taxa in the ocean (Breitbart, 2012; Pagarete et al., 2014).

### Empirical and *in silico* Abundance Comparison of HTVC010P and vSAG 37-F6 Viruses

Predominance of the isolate HTVC010P in Mediterranean Sea samples was revealed by virome fragment recruitment analysis (Figure 3). These results are consistent with the higher abundance of HTVC010P than vSAG 37-F6 obtained by ddPCR in both of our Western Mediterranean samples (BBMO and Cape Huertas). Thus, both bioinformatics and ddPCR data agreed on the predominance of phage HTVC010P in the studied samples and showed similar values of the relative abundances of both viruses, with log(HTVC010P/vSAG37-F6) values of 0.23 and 0.44 for ddPCR and viromic fragment recruitment, respectively (Figure 3). It is worth noting that from all *Tara* viromes, HTVC010P was more abundant precisely in two samples with contrasting Chl concentrations and environmental data: sample BC067 from Benguela current coastal upwelling with 1.55 mg of Chl/m^3^ and MS018 from the oligotrophic Mediterranean Sea with 0.05 mg of Chl/m^3^ (Brum et al., 2015). Indeed, relative abundances between HTVC010P and vSAG37-F6 did not significantly correlate with Chl content or other physico-chemical (e.g., nitrite, phosphate, or O_2_) and biological (e.g., total bacteria or cyanobacteria abundance) parameters from *Tara* expedition (Brum et al., 2015). Thus, data suggest that other –less apparent- environmental and biological factors would be determining the dynamics of these two pelagiphages and their specific *Pelagibacter* strain’s hosts.

### ddPCR Biases, a Critical Perspective: From Viral Purification and DNA Extraction to PCR Primer Design

Biases may occur at several steps during seawater sample processing (filtration–concentration–DNA purification and extraction) that could impact the final yield and quality of viral DNA for further molecular analyses and consequently, lead to inaccuracies in ddPCR quantification. For instance, filtration through 0.2 μm pore size filters and sample concentration by TFF can remove virus particles through retention onto the filters (due to binding to the membrane and/or removal of virus particles aggregates) or particle shearing (John et al., 2011; Cashdollar and Wymer, 2013; Hurwitz et al., 2013; Cai et al., 2015). The efficiency of these and other virus concentration and viral DNA extraction methods, such as iron chloride flocculation (John et al., 2011) is variable and it may depend on the viral load as well as the amount and nature of other organic and inorganic matter in the sample. In the ddPCR workflow, an efficient unbiased viral DNA extraction protocol is paramount. Different methods are currently being used in marine virology, such as commercial kits or phenol-based extractions (Espy et al., 2006; Bustin et al., 2009; Cook et al., 2009; Hurwitz et al., 2013; Koontz et al., 2015; Klenner et al., 2017). In general, it is assumed that they do not preferentially select some specific viruses to the detriment of others since in all cases the lysis mainly relies on proteinase K digestion to break the capsid. Due to the difficulty for estimating the efficiency of the viral DNA extraction, we have chosen to mathematically correct the absolute ddPCR abundance data by normalizing with the amount of viral DNA (ng) [see methods for details and Supplementary Material (excel file)]. With this method, regardless of the protocol employed to purify and extract the viral DNA, and the amount of DNA added per ddPCR reaction mix, data can be downstream normalized and corrected for cross-comparison between studies. Alternatively, albeit less accurate, data can be corrected instead, only taking into account the empirical viral loss factor value during the purification and concentration process (percentage of lost viruses during the ultraconcentration process: original viruses per mL/viruses per mL in the ultra concentrate sample). However, this method does not consider the differences in DNA extraction efficiencies. Nevertheless, as shown in Supplementary Material, both methods provide similar results.

Finally, the primer and probe design is another important step for obtaining accurate data from ddPCR. If a primer/probe is not specific to our desired target, the PCR could amplify DNA from undesired viruses that would lead to a clear overestimation of abundance data. Similarly, sub-estimation because of primer/probe mismatch against the target would lead to a sub-optimal amplification. The designed primers and probe for the vSAG 37-F6 was specific enough to allow discrimination against 7 other similar vSAG37-F6-like viruses reported in the literature (Labonté et al., 2015; Mizuno et al., 2016; Berube et al., 2018; Martinez-Hernandez et al., 2018). For vSAG 37-F6, only one contig assembled from *Tara* viromes (Coutinho et al., 2017) (ENA| FUIF01023531| FUIF01023531.1) was *in silico* found to match with our primers and probe set. This contig showed a very high nucleotide identity (94% identity) with vSAG 37-F6, indicating that both belonged to probably the same viral species (Figure 4B). However, when comparing the specific primers previously reported for the isolated pelagiphage HTVC010P, these also matched with other viral members non-specifically (*n* = 13; between 2,758 and 36,091 contig length) in databases with contrasting genome average nucleotide identity values; as low as 9% (Figure 4B). Both *in silico* and empirical data showed that other marine viruses shared an identical HTVC010P genomic region used for primer design in a previous study (Eggleston and Hewson, 2016). This likely explains the non-specific amplicon sequences obtained (Figure 4) and indicates that the currently published primers are no longer specific, after the recent expansion of viral databases. Although in our analyzed samples, as shown in Figure 4B, empirical Illumina sequencing data for HTVC010P showed that nearly 95% of all amplicons were specific and actually belonged to this virus, the bias and impact could be different for other environmental samples in which the abundance of these off-target viruses is higher. Nevertheless, our limits for *in silico* primer/probe design are determined by what we currently know about the marine virosphere and beyond that, nothing can be anticipated.

**FIGURE 5:**
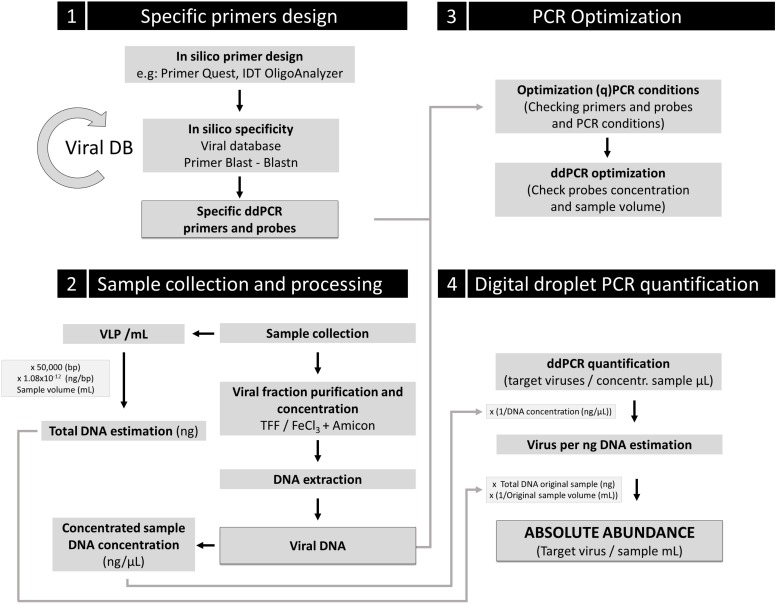
Proposal guideline for absolute viral quantification using ddPCR. Four key steps are indicated in the scheme. A comprehensive (and renewable) viral database for specific primers and probe design should be used. Control of inherent loss of VLP during sample processing should be implemented to correct the obtained absolute viral abundance for ddPCR quantification, using the loss factor relation. An additional control to determine the potential contamination in the viral fraction with infected cells for the target viruses is strongly recommended (e.g., DAPI or SYBR Gold staining of the viral fraction to ensure no presence of cells).

Based on this study and according to the expected feasibility and democratization of this technique in a near future, we propose that ddPCR could be a reference tool in marine virology to address absolute quantification of specific relevant viruses in environmental samples. Our data also underline the need of using, when possible, the same standardized protocol among laboratories (see our proposal in Figure 5) in order to build biological meaningful datasets for further cross-comparisons. We also would like to emphasize special attention to the design of primers and probes, a critical step of the pipeline.

Thus, here we show that the application of ddPCR to natural samples can be a robust approach to provide accurate estimates of absolute abundance of relevant uncultured viruses in the marine viriosphere. This novel methodology applied to marine viral ecology will aid to unveil viral patterns and dynamics linked to their hosts and even their contribution to biogeochemical cycles. In the years ahead, as more data are collected from different environmental samples and viruses, it is likely that our knowledge on the “viral shunt” and the viral contribution to the global biogeochemical cycles will be closer to reality.

## Data Availability

The datasets generated for this study can be found in https://de. cyverse.org/dl/d/773F61F8-65B4-4D20-9E59-D9C0EF163176/Da tabase_Viral_genomes_Martinez-Hernandez_Frontiers.fasta.gz.

## Author Contributions

MM-G has conceived, designed and supervised the experiments, provided funds, analyzed data, and wrote the manuscript. FM-H has designed and conducted experiments, analyzed data and wrote the manuscript. IG-H, MG, and LM-C have performed experiments and analyzed data. JMM has designed and conducted experiments, analyzed data, provided funds, and wrote the manuscript.

## Conflict of Interest Statement

The authors declare that the research was conducted in the absence of any commercial or financial relationships that could be construed as a potential conflict of interest.
