# Supplementary material for: Droplet Digital PCR for Estimating Absolute Abundances of Widespread Pelagibacter Viruses
Source: Front Microbiol. 2019 Jun 12;10:1226. doi: 10.3389/fmicb.2019.01226 (PMC6581686; doi:10.3389/fmicb.2019.01226)
Supplement: Supplementary file 1 [file Data_Sheet_1.ZIP › Supplementary Material/Data sheet 2.docx]

https://de.cyverse.org/dl/d/773F61F8-65B4-4D20-9E59-D9C0EF163176/Database_Viral_genomes_Martinez-Hernandez_Frontiers.fasta.gz
