# Supplementary material for: Droplet Digital PCR for Estimating Absolute Abundances of Widespread Pelagibacter Viruses
Source: Front Microbiol. 2019 Jun 12;10:1226. doi: 10.3389/fmicb.2019.01226 (PMC6581686; doi:10.3389/fmicb.2019.01226)
Supplement: Supplementary file 1 [file Data_Sheet_1.ZIP › Supplementary Material/Supplementary Material Figures 1-4_Martinez-Hernandez.docx]

Supplementary Material

**Droplet digital PCR for estimating absolute abundances of widespread Pelagibacter viruses**

**Francisco Martinez-Hernandez^1^, Inmaculada Garcia-Heredia^1^, Monica Lluesma Gomez, Lucia Maestre-Carballa^1^, Joaquín Martínez-Martínez^2^, and Manuel Martinez-Garcia^1*^**

^1^Department of Physiology, Genetics, and Microbiology, University of Alicante. Alicante, Spain.

^2^Marine Virology Laboratory, Bigelow Laboratory for Ocean Sciences, East Boothbay, ME, USA

*** Correspondence:**Manuel Martinez-Garcia
m.martinez@ua.es

This supplementary material contains 4 figures


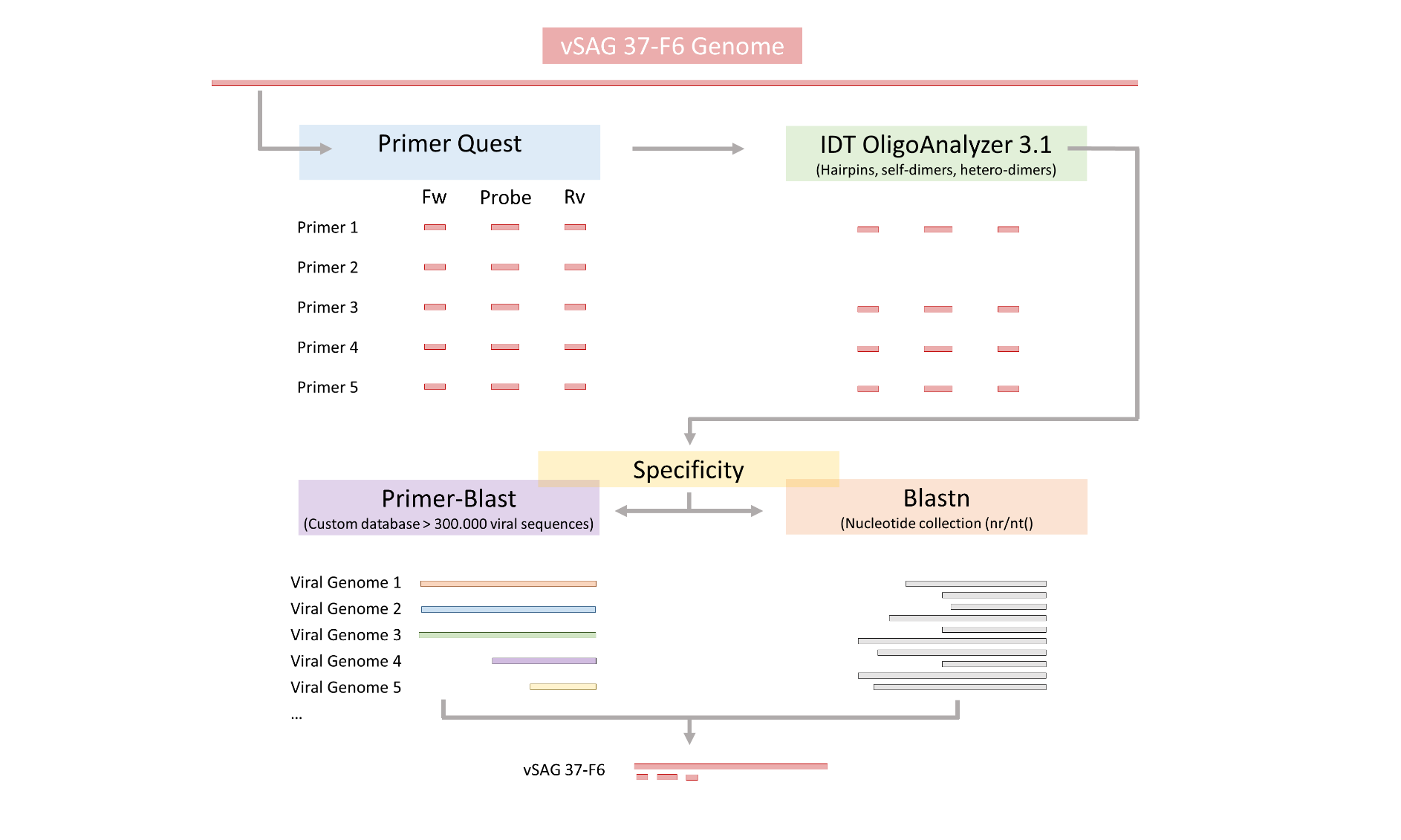
**Supplementary Figure 1.** Schematic representation of the specific primer set and probe design. First (Primer Quest and IDT Oligoanalizer steps) are used to find primers and probes of the target viral sequence with the desired parameters (Tm, Length, %GC, etc…). Then specificity is checked comparing primers and probe with a custom viral database, and NCBI nucleotide collection (nt/nr).


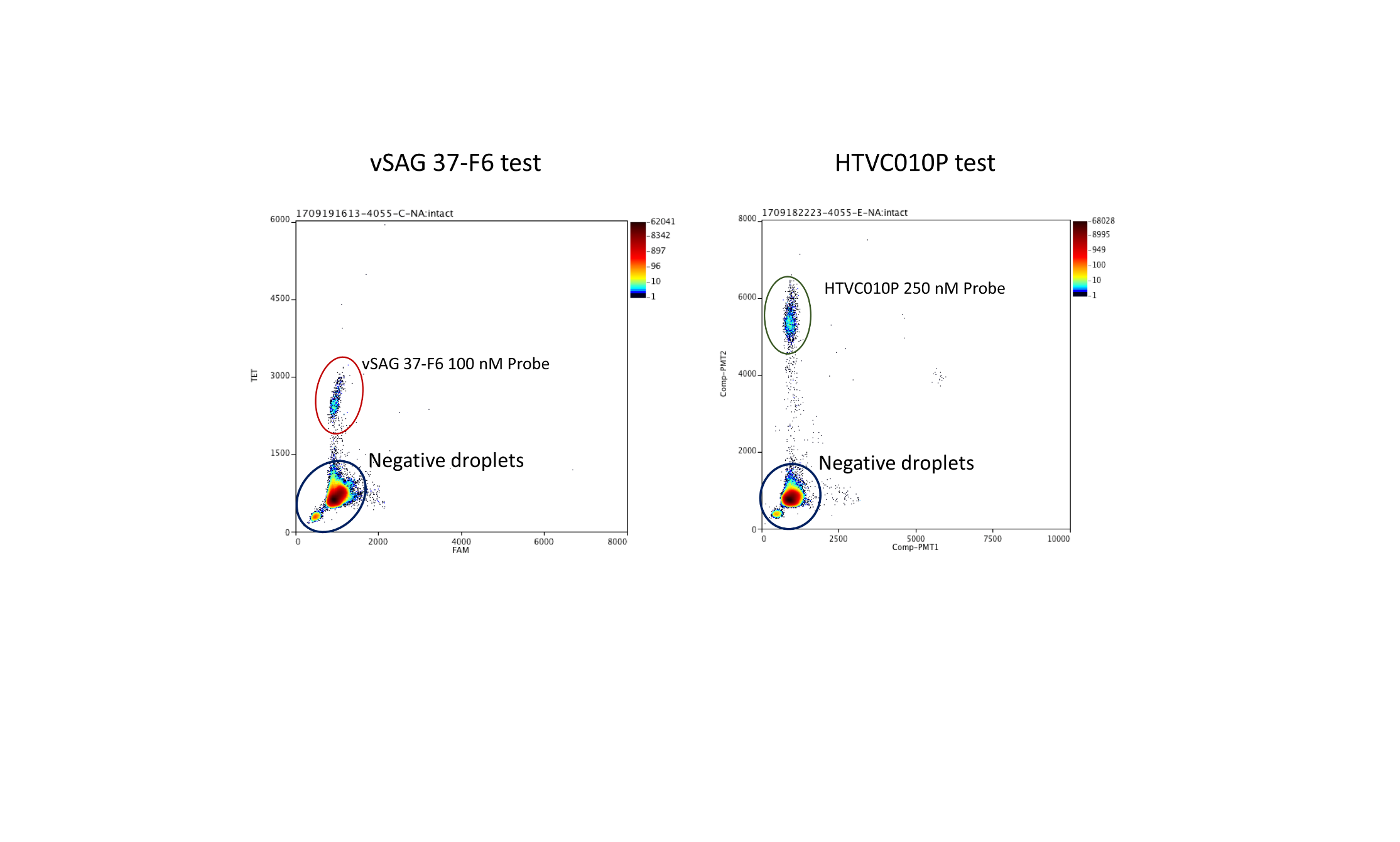


**Supplementary Figure 2.** **ddPCR probe concentration optimization.** Experiments with Taqman probe for vSAG 37-F6 and HTVC010P individually tested using PCR product of each target DNA, to determine optimum concentration and location in the ddPCR plot.


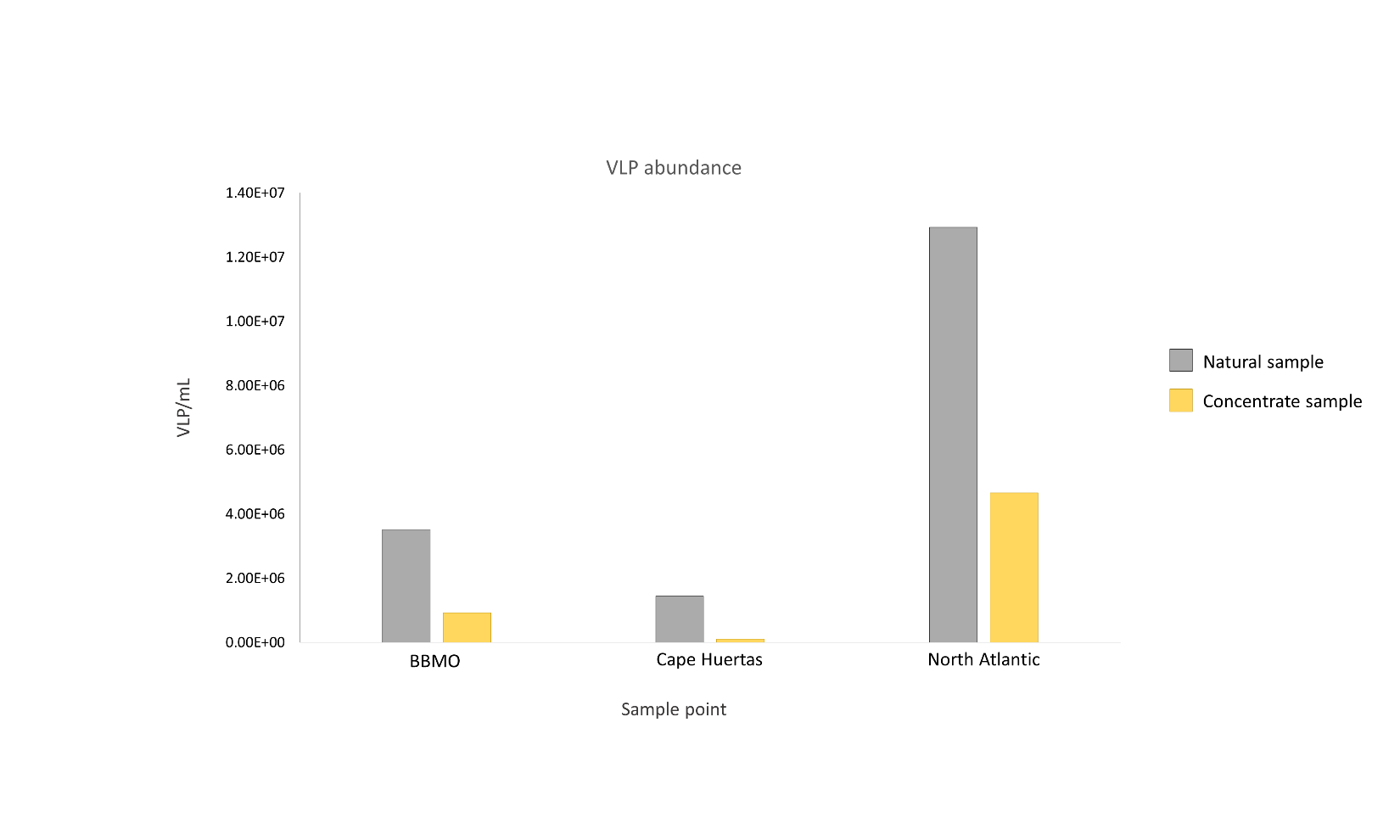


**Supplementary Figure 3.** VLP abundances in natural and concentrated samples as estimated by flow cytometry. Consistent removal of virus particles in all samples during the process to produce a concentrate is evident from this result.

**
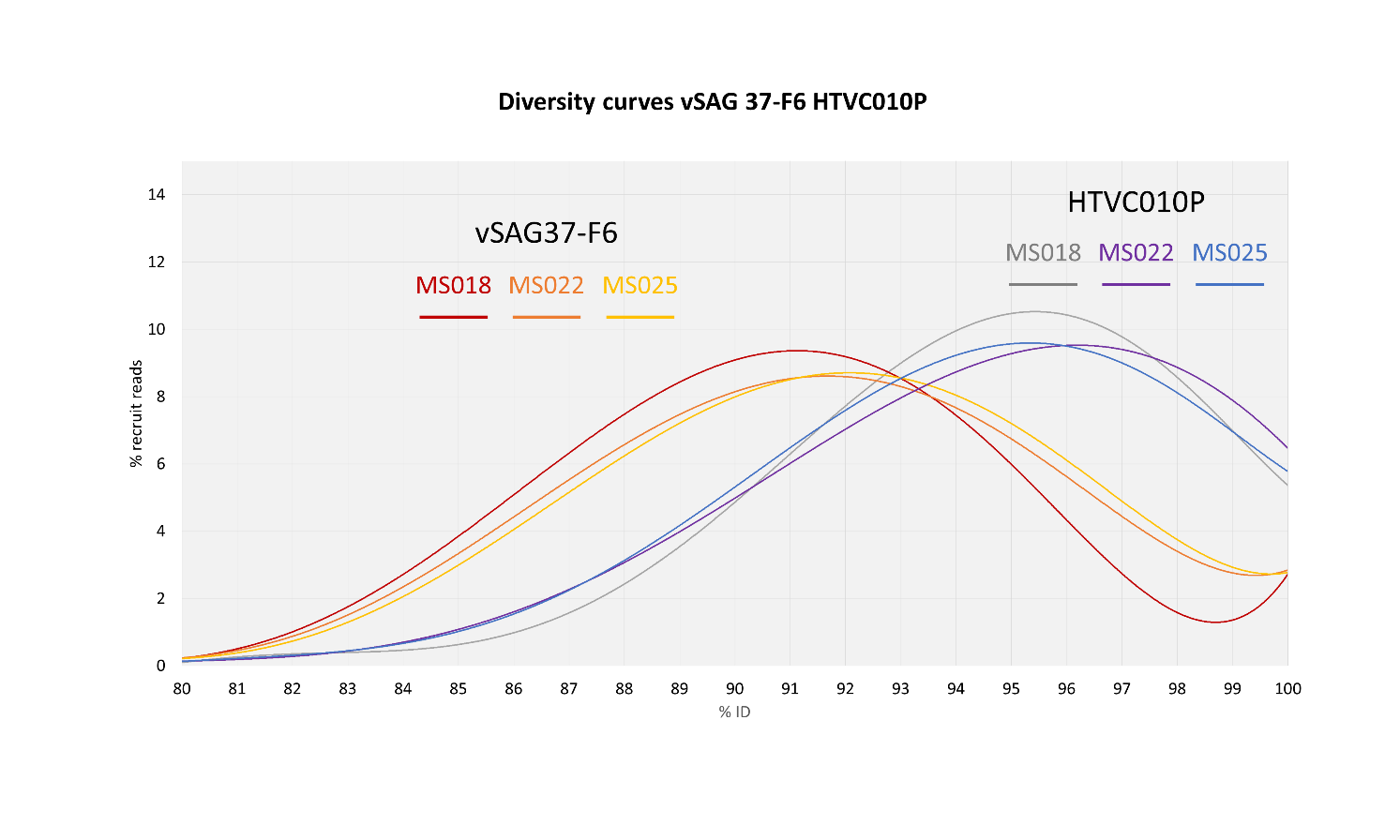
**

**Supplementary Figure 4. Diversity curves of vSAG 37-F6 and HTVC010P.** Distribution of identity of recruited reads by each virus is shown using tree different *Tara* viromes from Mediterranean Sea (MS018, MS022, and MS025 from the Mediterranean Sea). The soft slope and the peak near to 90% of identity indicate a high microdiversity (see previous study by Martinez-Hernandez et al 2017).
